# Rate Pressure Products Affect the Relationship between the Fractional Flow Reserve and Instantaneous Wave-Free Ratio

**DOI:** 10.1155/2020/6230153

**Published:** 2020-07-21

**Authors:** Suguru Ebihara, Hisao Otsuki, Hiroyuki Arashi, Junichi Yamaguchi, Nobuhisa Hagiwara

**Affiliations:** Department of Cardiology, The Heart Institute of Japan, Tokyo Women's Medical University, Tokyo, Japan

## Abstract

The rate pressure product (RPP) is an index of myocardial metabolism that correlates closely with myocardial hemodynamics. The relationship between the RPP and the fractional flow reserve (FFR) and instantaneous wave-free ratio (iFR) is not known. In this study, we investigated the effects of the RPP on the FFR and iFR. We retrospectively enrolled 195 patients (259 lesions) who had undergone invasive coronary angiography and both the iFR and FFR examinations between 2012 and 2017. The RPP was defined as systolic blood pressure multiplied by the heart rate, measured prior to the iFR evaluation. The study population was divided into the low-RPP (*n* = 129, mean RPP: 6981 ± 1149) and high-RPP (*n* = 130, mean RPP: 10391 ± 1603) groups according to the median RPP. Correlations and biases between the iFR and FFR were compared. The diagnostic performance of the iFR in the groups was calculated, using FFR as the gold standard. The correlation between the iFR and FFR was higher in the high-RPP group than in the low-RPP group. The bias between the iFR and FFR in the high-RPP group was smaller than that in the low-RPP group. The best cutoff value of the iFR for predicting an FFR of 0.8 was 0.90 for all lesions, 0.93 for the low-RPP group, and 0.82 for the high-RPP group. The iFR and RPP showed a weak but a statistically significant negative correlation (*R* = 0.14; *p* = 0.029). This was not observed for the relationship between the FFR and RPP. In conclusion, the RPP affects the relationship between the FFR and iFR. With FFR as the gold standard, the iFR may underestimate and overestimate the functionality of ischemia in the low- and high-RPP groups, respectively.

## 1. Introduction

Several studies have shown that reducing myocardial ischemia with coronary artery intervention improves both quality of life and clinical outcomes [1, 2]. The fractional flow reserve (FFR) is a hyperemic pressure-derived ratio that is considered the reference standard method for evaluating the functional severity of coronary artery stenosis based on substantial clinical outcome data [3–5]. The instantaneous wave-free ratio (iFR) is a nonhyperemic pressure-derived ratio; randomized controlled trials have demonstrated that the iFR-guided revascularization is not inferior to FFR-guided revascularization [6, 7].

The FFR and iFR correlate well and are similar coronary functional indexes, although they differ in some respects; specifically, FFR is calculated by the pressure ratio during the entire cardiac cycle period under hyperemia, while the iFR is calculated by the pressure ratio during the diastolic cardiac cycle under resting conditions. The rate pressure product (RPP) is calculated as systolic blood pressure multiplied by the heart rate, which is an index of myocardial metabolism that is closely correlated with myocardial hemodynamics [8]. Previous reports suggested that the coronary blood flow at resting condition was easily affected by the fluctuation in blood pressure or heart rate, but the coronary blood flow under hyperemia was not [9, 10]. No studies, to date, have reported on the effects of the RPP on the relationship between the FFR and iFR. Therefore, the aim of this study was to evaluate the effects of the RPP on the FFR and iFR relationship. We also determined how the diagnostic performance of the iFR is affected by the RPP when FFR is set as the gold standard.

## 2. Materials and Methods

### 2.1. Patient Population

In this study, we enrolled patients who had undergone clinically indicated invasive coronary angiography as well as both the iFR and FFR examinations between 2012 and 2017. Because we examined how the RPP affect the relationship between the FFR and iFR, we limited our analysis to patients who had undergone blood pressure and heart rate measurements just prior to the iFR and FFR evaluations. It is difficult to accurately assess the RPP in patients with atrial fibrillation. We, therefore, excluded patients who had had atrial fibrillation. Hemodialysis patients characteristically exhibit specific hemodynamic conditions; therefore, such patients were excluded. We also excluded patients with lesions in the left main trunk and those with bypass grafts. Finally, we excluded patients with ST segment elevation myocardial infarction, non-ST-segment elevation myocardial infarction, or New York Heart Association class IV heart failure. We included the patients with unstable angina pectoris, but the stenoses interrogated were the nonculprit lesions.

### 2.2. Measurement of Rate Pressure Products

The RPP was defined as the systolic blood pressure multiplied by the heart rate. Blood pressure and heart rate measurements taken just prior to the iFR measurement were used for the calculation. Blood pressure data were extracted from hemodynamic records taken during cardiac catheterization and had been measured using either invasive monitoring of the arterial catheter or a sphygmomanometer. Heart rate was recorded from the electrocardiography monitor or the oxygen saturation monitor. We divided lesions into two groups based on whether the RPP just prior to the iFR measurement for each lesion was greater or lesser than the median RPP; thus, patients in the low- and high-RPP groups had lesions with the RPP <8512 and ≥8512, respectively. Lesion characteristics were compared between the groups as were the correlations and biases between the FFR and iFR.

### 2.3. Coronary Angiography, Quantitative Coronary Angiography, and Echocardiography

Coronary angiography was performed according to standard clinical methods via the radial or femoral arterial approach. Quantitative coronary angiography (QCA) was performed by an independent physician using a computer-assisted automated edge detection algorithm [11]; the physician was blinded to the results of the iFR and FFR. The external diameter of the contrast-filled catheter (5-Fr or 6-Fr) was used as the calibration standard. The percentage of the stenosis diameter during end-diastole was measured using the worst-view trace. Echocardiography measurements were performed according to American Society of Echocardiography guidelines by an independent physician who was blinded to the results of the FFR and iFR. The left ventricular mass index and *E*/*e*ʹ ratio were added to the analysis.

### 2.4. Standard iFR and FFR Measurements

Both the iFR and FFR examinations were performed using either diagnostic or interventional guiding catheters. After administering an intracoronary bolus of nitroglycerin, a coronary pressure wire (Prime Wire Prestige; Philips Volcano Corporation, San Diego, CA, USA) was calibrated outside of the body and advanced such that the sensor was positioned at the tip of the guiding catheter where the two pressures were equalized and recorded. After completion of the pressure equalization at the tip of the guide catheter, the guidewire was advanced to a point distal to the stenosis. First, the iFR was directly and automatically measured online using the Volcano Core system (Philips Volcano). Second, the FFR was measured during maximal hyperemia. Hyperemia in the target coronary artery was achieved either with an intracoronary bolus injection of 8–12 mg papaverine or with continuous intravenous administration of adenosine at 150 *µ*g/kg/min. At the end of each measurement, the pressure sensor was retracted to the tip of the guide catheter to avoid pressure drift.

### 2.5. Statistical Analysis

Continuous data were expressed as means ± standard deviations. Categorical data were expressed as absolute values and percentages. The comparisons were made using Welch's *t*-test for normally distributed continuous variables, the Mann–Whitney *U* test for nonnormally distributed continuous variables, and Pearson's chi-squared test for categorical variables. Correlations between parameters were tested using Pearson's correlation coefficients. Fisher's r-to-z transformation was used to assess the significance of the difference between two correlation coefficients. Bland–Altman analysis was conducted to evaluate the bias and limits of agreement between each parameter. ROC curves were used to evaluate the diagnostic performance of the iFR when identifying a positive FFR measurement using the area under the curve (AUC). Multivariable regression analysis was performed to determine predictors of the FFR and iFR. Variables were included in the multivariable model if they reached *p* < 0.20 after univariable regression analysis. *p* values <0.05 were considered to indicate statistical significance. Statistical analyses were performed using JMP statistical software (JMP Pro 14.0; SAS Institute Inc., Cary, NC, USA).

### 2.6. Compliance with Ethical Standards

The study protocol was based on the regulations of the hospital's ethics committee. All participating patients provided written informed consent. The study was conducted according to the principles of the 1975 Declaration of Helsinki.

## 3. Results

We enrolled 195 consecutive patients with 259 lesions in this study. The study patient characteristics are shown in Table 1. The mean patient age was 68.8 ± 10.4 years; 57.9% of patients had diabetes mellitus, 72.3% had hypertension, and 67.2% had hypercholesterolemia. The mean left ventricular ejection fraction was 51.3%, and the mean glomerular filtration rate was 60.4 mL/min/1.73 m^2^.

The median RPP was 8512 (interquartile range: 7200, 10220) (Supplemental Figure 1). A total of 129 (49.8%) lesions were classified as belonging to the low-RPP group (mean RPP: 6981 ± 1149) and 130 (50.2%) to the high-RPP group (mean RPP: 10391 ± 1603) according to the median RPP. Lesion characteristics of the low-RPP and high-RPP groups are shown in Table 2.

In comparison with the lesions in the low-RPP group, the lesions in the high-RPP group tended to be in older patients, females, nonsmokers, and patients with a history of myocardial infarction. The E/e' ratio in the high-RPP group was higher than that in the low-RPP group (14.1 ± 6.4 vs. 12.5 ± 4.5, respectively, *p*=0.045). No significant difference was observed in the QCA parameters.

**Figure 1:**
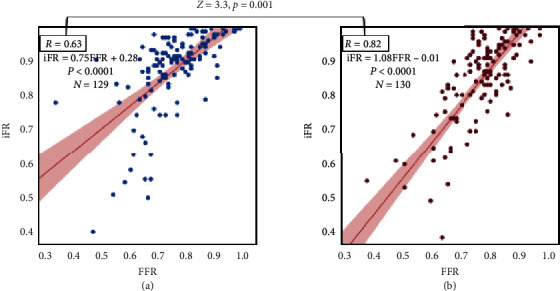
Scatter plots of the FFR and iFR in the low-RPP group (a) and the high-RPP group (b). RPP, rate pressure product; iFR, instantaneous wave-free ratio; FFR, fractional flow reserve.

The correlation between the FFR and iFR in the high-RPP group was significantly higher than that of the low-RPP group (Pearson's correlation: *r* = 0.82 vs. *r* = 0.63, respectively, *z* = 3.3, *p*=0.001, Figure 1).

**Figure 2:**
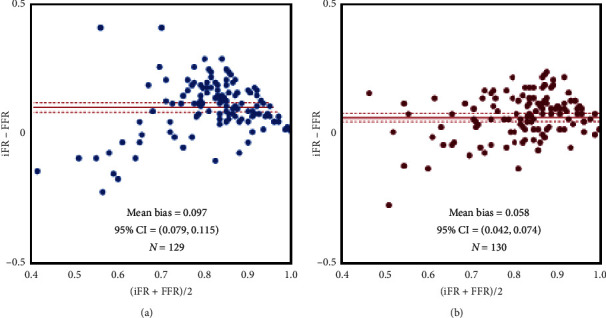
Bland–Altman plot comparing the FFR and iFR in the low-RPP group (a) and the high-RPP group (b). RPP, rate pressure products; iFR, instantaneous wave-free ratio; FFR, fractional flow reserve.

According to Bland–Altman analysis, the bias between the FFR and iFR in the high-RPP group was 0.058 (95% confidence interval (CI): 0.042–0.074) and that of the low-RPP group was 0.097 (95% CI: 0.079–0.115) (Figure 2).

**Figure 3:**
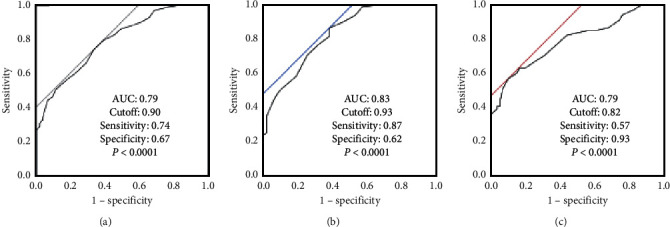
Receiver-operating characteristic curves of the iFR values for an FFR of 0.8 in all lesions (a), the low-RPP group (b), and high-RPP group (c). RPP, rate pressure products; AUC, area under the curve.

The best cutoff value of the iFR for predicting an FFR of 0.80 was 0.90 for all lesions (AUC 0.79, sensitivity 0.74, specificity 0.67, and *p* < 0.0001), with 0.93 for the low-RPP group (AUC 0.83, sensitivity 0.87, specificity 0.62, and *p* < 0.0001), and 0.82 for the high-RPP group (AUC 0.79, sensitivity 0.57, specificity 0.93, and *p* < 0.0001) (Figure 3) (Supplemental Figure 2).

Using the current iFR cutoff values of ≤0.89, 26.4% of lesions in the low-RPP group would be underestimated, while 16.9% of lesions in the high-RPP group would be overestimated (Supplemental Figure 3).

**Figure 4:**
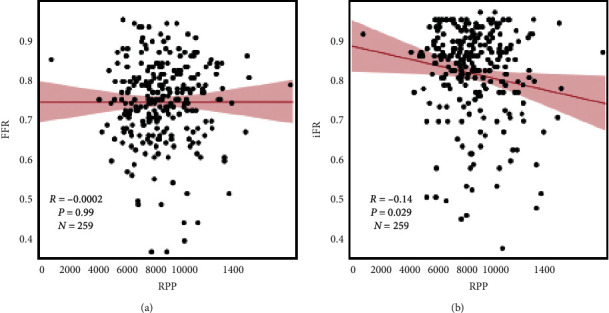
Scatter plots comparing the RPP with the FFR (a) and iFR (b). RPP, rate pressure products; iFR, instantaneous wave-free ratio; FFR, fractional flow reserve.

Though there was no significant correlation between the RPP and FFR, the RPP and iFR showed a weak but significant inverse correlation (Pearson's correlation: *r* = 0.14; *p*=0.029) (Figure 4).

Table 3 shows the variables which were associated with the iFR or FFR. In the univariable analysis, female sex (*p*=0.11) and the presence of diabetes mellitus (*p*=0.04) were potential predictors of FFR. The age (*p*=0.02), presence of diabetes mellitus (*p* < 0.0001), prior revascularization (*p*=0.05), left ventricular mass index (*p*=0.19), E/e' (*p*=0.01), glomerular filtration rate (*p*=0.01), and RPP (*p*=0.04) were potential predictors of the iFR. Presence of diabetes mellitus was the independent predictor of FFR in multivariable analysis (*p*=0.04; *β* = 0.13). The presence of diabetes mellitus (*p*=0.002; *β* = 0.23) and the RPP (*p*=0.04; *β* = -0.15) were independent predictors of the iFR.

**Figure 5:**
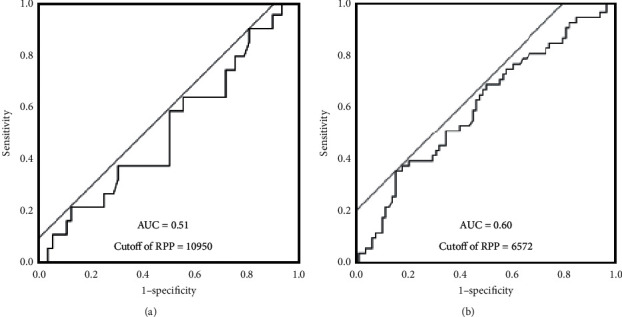
The best cutoff value of rate pressure products to predict discordance of the iFR ≤ 0.89 and FFR > 0.8 (a) and discordance of the iFR > 0.89 and FFR ≤ 0.8 (b). RPP, rate pressure products; iFR, instantaneous wave-free ratio; FFR, fractional flow reserve.

The best cutoff value of the RPP to predict discordance of the iFR ≤ 0.89 and FFR > 0.8 was 10950 and 6572 for discordance of the iFR > 0.89 and FFR ≤ 0.8 (Figure 5).

## 4. Discussion

The RPP is an index that reflects myocardial metabolism and greatly affects the hemodynamics of the heart [8], suggesting that coronary artery pressure-derived indexes could be influenced by the RPP. No previous study has reported the influence of the RPP on the relationship between the FFR and iFR.

The primary findings in the present study were as follows: (1) the correlation between the FFR and iFR in the high-RPP group was higher than that of the low-RPP group; (2) the best cutoff value of the iFR for predicting an FFR of 0.8 was 0.90 for all lesions, 0.93 for the low-RPP group, and 0.82 for the high-RPP group; and (3) the iFR and RPP showed a weak but a statistically significant negative correlation. No similar result was found for the FFR and RPP. Moreover, multivariable analysis revealed that the RPP was independently associated with the iFR.

The reason for the higher correlation between the iFR and FFR in the high-RPP group might be that the oxygen consumption of the myocardium was increased, resulting in increased coronary blood flow to maintain the oxygen supply in the high-RPP group. If one simply considers the amount of blood flow in the coronary arteries, coronary blood flow under resting conditions in the high-RPP group may increase and be closer to that of the hyperemic condition. Hence, the correlation between the iFR and FFR in the high-RPP group is likely to be increased. The question remains as to whether FFR would be affected in a high-RPP environment. A previous study reported that coronary blood flow under hyperemia did not correlate with the RPP, while coronary blood flow under resting conditions had a significant positive correlation with the RPP [9]. Similarly, although we did not observe a significant correlation between the RPP and FFR, the RPP and iFR showed a weak but a significant negative correlation. de Bruyne et al. reported that the FFR was almost independent of hemodynamic changes including heart rate and blood pressure [10]. Kolli et al. reported that fluctuations in the heart rate had no significant influence on the measured values of FFR in a porcine model [12]. By contrast, a study reported that hyperemic coronary flow decreased, and resting coronary flow was maintained in an environment where coronary circulation compensated for microvascular resistance [13]. However, if the FFR is measured when the coronary microcirculation is optimally dilated using the correct method and correct hyperemic agent, the FFR value might not be affected by the RPP value.

As regards the patient characteristics in our study, a total of 58% patients had diabetes mellitus, which is an important covariate for microvascular dysfunction. There are also numerous reports which have suggested that the higher left ventricular mass index and higher *E*/*e*′ ratio are associated with microvascular dysfunction [14, 15]. When adding the variables including the presence of diabetes mellitus, left ventricular mass index, and E/e' ratio to the multivariable analysis to clarify the predictor of the iFR, the RPP was independently associated with the iFR. These data suggest that the iFR might be affected by the RPP regardless of microvascular dysfunction.

In this study, the best cutoff value of the iFR for predicting an FFR of 0.8 was 0.90 for all lesions. This value agrees with previously reported values. The best cutoff value for the low-RPP group was 0.93, higher than the standard predictive value of the iFR. The best cutoff value for the high-RPP group was 0.82, lower than the standard predictive value of the iFR. When using the corrected iFR cutoff values of ≤0.93 for the low-RPP group and ≤0.82 for the high-RPP group, a total of 23 (17.8%) lesions should be reclassified as “ischemic” in the low-RPP group and a total of 16 (12.3 %) lesions should be reclassified as “nonischemic” in the high-RPP group.

The FFR has hemodynamic independence through dilating and maximizing the coronary microcirculation in hyperemia. The iFR value is sensitive to the RPP fluctuations, while the FFR is less susceptible to the RPP fluctuations. Jain et al. reported that mental stress increased the RPP [16]. In this study, the stressful condition may have the greatest effect on the RPP value because the comorbidity of hypertension did not change between the high-RPP group and the low-RPP group. When examining the iFR, it may also be necessary to make this as stress-free as possible. Then, the difference between low- vs. high-RPP is more likely associated with patient hemodynamics at the time of physiologic assessment, further suggesting the stability of hyperemic measurements as opposed to resting measurements. Even if the iFR is positive, there is a possibility of false positive if the RPP is 10950 or more at the time of the iFR measurement. Further, if the iFR is negative, there is a possibility of false negative if the RPP is 6572 or less at the time of the iFR measurement. Additional studies with a prospective study design and larger numbers of patients are necessary to validate the relation between the rate pressure products and iFR and FFR.

There are several limitations to this study. First, this was a retrospective observational cohort study conducted at a single center, and the number of study patients was relatively small. Second, there were no data on the changes in the iFR values at rest and during exercise in the same patients, and there were also no data on the clinical endpoints. Third, the prevalence of diabetes mellitus and hypertension was higher than those of previous studies. These comorbidities induce structural changes in the myocardium and reduce coronary capacity, possibly influencing the relationship between the iFR and FFR. Fourth, analyses vary depending on which indicator is considered the gold standard. There is, however, abundant evidence in favor of using the FFR, and it is currently the most reliable index recommended in the guidelines. Furthermore, the FFR has customarily been the benchmark for evaluating other nonhyperemic indices. Fifth, there are no data on medication (i.e., calcium-channel blocker and beta-blocker), which might influence the results.

## 5. Conclusion

Rate pressure products may affect the relationship between the FFR and iFR. Setting FFR as the reference gold standard, the iFR may underestimate the functionality of ischemia in the low-RPP group and overestimate it in the high-RPP group.

## Figures and Tables

**Table 1:** Patients' characteristics.

Patients' variables	*n* = 195
Age	68.8 ± 10.4
Men	45 (23.1%)
Body mass index (kg/m^2^)	23.9 ± 3.5
Diabetes mellitus	113 (57.9%)
Hypertension	141 (72.3%)
Hyperlipidemia	131 (67.2%)
Smoking	69 (35.4%)
Prior myocardial infarction	58 (29.7%)
Revascularization	105 (53.8%)
Left ventricular ejection fraction (%)	51.3 ± 9.7
Chronic kidney disease (>stage II)	102 (52.3%)
Glomerular filtration rate (mL/min/1.73 m^2^)	60.4 ± 16.4

^*∗*^Data are expressed as mean ± standard deviation or as number (percentage).

**Table 2:** Lesion characteristics divided by the median of the rate pressure products.

	Low-RPP group (*n* = 129)	High-RPP group (*n* = 130)	*p* value
Age	67.8 ± 11.4	70.8 ± 9.0	0.02
Female	23 (17.8%)	40 (30.8%)	0.02
Body mass index (kg/m^2^)	23.7 ± 3.8	23.9 ± 3.6	0.74
Diabetes mellitus	68 (52.7%)	83 (63.9%)	0.08
Hypertension	92 (71.3%)	98 (75.4%)	0.48
Hyperlipidemia	95 (73.6%)	83 (63.9%)	0.11
Smoking	59 (45.7%)	37 (28.5%)	0.005
Prior myocardial infarction	33 (25.6%)	49 (37.7%)	0.045
Revascularization	78 (60.5%)	68 (52.3%)	0.21
LVEF (%)	49.9 ± 10.6	51.9 ± 8.7	0.10
LVMI (g/m^2^)	98.7 ± 29.8	91.5 ± 30.6	0.11
*E*/*e*′	12.5 ± 4.5	14.1 ± 6.4	0.048
Chronic kidney disease (>stage II)	69 (53.5%)	71 (54.6%)	0.9
GFR (mL/min/1.73 m^2^)	60.9 ± 17.1	58.0 ± 17.2	0.18
Percent diameter stenosis	63.6 ± 17.4	63.0 ± 18.3	0.77
Left anterior descending artery	78 (60.5%)	69 (53.1%)	0.26
Circumflex artery	24 (18.6%)	30 (23.1%)	0.44
Right coronary artery	27 (20.9%)	31 (23.9%)	0.66
FFR	0.77 ± 0.11	0.78 ± 0.12	0.51
iFR	0.87 ± 0.13	0.84 ± 0.16	0.09
Percent diameter stenosis	64 ± 17	63 ± 18	0.77
Lesion diameter (mm)	17.7 ± 6.9	18.5 ± 6.9	0.32
Reference diameter (mm)	2.7 ± 0.5	2.6 ± 0.5	0.06
Diffuse/tandem lesion	37 (28.7%)	37 (28.7%)	0.89
Rate pressure products	6981 ± 123	10392 ± 122	<0.0001
Systolic blood pressure (mmHg)	111 ± 19.0	138 ± 19.9	<0.0001
Heart rate (beats/minute)	63.3 ± 8.4	76.0 ± 12.2	<0.0001

RPP, rate pressure products; LVEF, left ventricular ejection fraction; LVMI, left ventricular mass index; GFR, glomerular filtration rate; FFR, fractional flow reserve; iFR, instantaneous wave-free ratio. ^*∗*^Data are expressed as mean ± standard deviation or as number (percentage).

**Table 3:** The variables associated with the FFR and iFR.

	FFR				iFR			
	Univariable		Multivariable		Univariable		Multivariable	
	*β*	*p* value	*β*	*p* value	*β*	*p* value	*β*	*p* value
Age	0.04	0.52			−0.15	0.02	−0.01	0.87
Female	−0.1	0.11	−0.1	0.12	0.03	0.59		
Body mass index	−0.07	0.25			0.02	0.77		
Diabetes mellitus	0.13	0.04	0.13	0.04	0.24	<0.0001	0.23	0.002
Hypertension	0.005	0.94			0.02	0.7		
Dyslipidemia	0.03	0.61			−0.07	0.24		
Smoking	0.07	0.28			−0.05	0.47		
Prior myocardial infarction	−0.04	0.50			0.02	0.77		
Prior revascularization	−0.07	0.29			−0.12	0.05	−0.1	0.21
LVEF	0.02	0.79			0.04	0.52		
LVMI	−0.04	0.60			−0.1	0.19	−0.06	0.49
*E*/*e*′	−0.07	0.35			−0.18	0.01	−0.05	0.58
GFR	0.08	0.21			0.16	0.01	0.13	0.13
Rate pressure products	−0.001	0.99			−0.14	0.03	−0.15	0.04

LVEF, left ventricular ejection fraction; LVMI, left ventricular mass index; GFR, glomerular filtration rate; FFR, fractional flow reserve; iFR, instantaneous wave-free ratio.

## Data Availability

Clinical data used to support the findings of this study are restricted by the Tokyo Women's Medical University (TWMU) Ethics Committee in order to protect patient privacy. Data are available from the clinical research support center TWMU for researchers who meet the criteria for access to confidential data. Researchers can contact the corresponding author by e-mail.

## Supplementary Materials

Supplementary Materials Supplemental Figure 1: distribution of the rate pressure products. Supplemental Figure 2: diagnostic ability of the iFR in the high-RPP and low-RPP groups with respect to the FFR. Supplemental Figure 3: correlation between the FFR and iFR in the high-RPP and low-RPP groups.

## References

[1] Hachamovitch R., Hayes S. W., Friedman J. D., Cohen I., Berman D. S. (2003). Comparison of the short-term survival benefit associated with revascularization compared with medical therapy in patients with no prior coronary artery disease undergoing stress myocardial perfusion single photon emission computed tomography. *Circulation*.

[2] Shaw L. J., Berman D. S., Maron D. J. (2008). Optimal medical therapy with or without percutaneous coronary intervention to reduce ischemic burden: results from the Clinical Outcomes Utilizing Revascularization and Aggressive Drug Evaluation (COURAGE) trial nuclear substudy. *Circulation*.

[3] Tonino P. A. L., De Bruyne B., Pijls N. H. J. (2009). Fractional flow reserve versus angiography for guiding percutaneous coronary intervention. *New England Journal of Medicine*.

[4] De Bruyne B., Pijls N. H. J., Kalesan B. (2012). Fractional flow reserve-guided PCI versus medical therapy in stable coronary disease. *New England Journal of Medicine*.

[5] De Bruyne B., Fearon W. F., Pijls N. H. J. (2014). Fractional flow reserve-guided PCI for stable coronary artery disease. *New England Journal of Medicine*.

[6] Davies J. E., Sen S., Dehbi H. M. (2017). Use of the instantaneous wave-free ratio or fractional flow reserve in PCI. *The New England Journal of Medicine*.

[7] Götberg M., Christiansen E. H., Gudmundsdottir I. J. (2017). Instantaneous wave-free ratio versus fractional flow reserve to guide PCI. *New England Journal of Medicine*.

[8] Gobel F. L., Norstrom L. A., Nelson R. R., Jorgensen C. R., Wang Y. (1978). The rate pressure product as an index of myocardial oxygen consumption during exercise in patients with angina pectoris. *Circulation*.

[9] Czernin J., Müller P., Chan S. (1993). Influence of age and hemodynamics on myocardial blood flow and flow reserve. *Circulation*.

[10] de Bruyne B., Bartunek J., Sys S. U., Pijls N. H. J., Heyndrickx G. R., Wijns W. (1996). Simultaneous coronary pressure and flow velocity measurements in humans. *Circulation*.

[11] Suzuki N., Asano T., Nakazawa G. (2020). Clinical expert consensus document on quantitative coronary angiography from the Japanese Association of Cardiovascular Intervention and Therapeutics. *Cardiovascular Intervention and Therapeutics*.

[12] Kolli K. K., Banerjee R. K., Peelukhana S. V. (2011). Influence of heart rate on fractional flow reserve, pressure drop coefficient, and lesion flow coefficient for epicardial coronary stenosis in a porcine model. *American Journal of Physiology-Heart and Circulatory Physiology*.

[13] Nijjer S. S., de Waard G. A., Sen S. (2016). Coronary pressure and flow relationships in humans: phasic analysis of normal and pathological vessels and the implications for stenosis assessment: a report from the Iberian-Dutch-English (IDEAL) collaborators. *European Heart Journal*.

[14] Taqueti V. R., Solomon S. D., Shah A. M. (2018). Coronary microvascular dysfunction and future risk of heart failure with preserved ejection fraction. *European Heart Journal*.

[15] Arashi H., Yamaguchi J., Ri T. (2018). The impact of tissue Doppler index E/e′ ratio on instantaneous wave-free ratio. *Journal of Cardiology*.

[16] Jain D., Shaker S. M., Burg M., Wackers F. J. T., Soufer R., Zaret B. L. (1998). Effects of mental stress on left ventricular and peripheral vascular performance in patients with coronary artery disease. *Journal of the American College of Cardiology*.

