# Supplementary figures and images for: Rate Pressure Products Affect the Relationship between the Fractional Flow Reserve and Instantaneous Wave-Free Ratio
Source: J Interv Cardiol. 2020 Jul 21;2020:6230153. doi: 10.1155/2020/6230153 (PMC7396073; doi:10.1155/2020/6230153)

Distribution of rate-pressure products

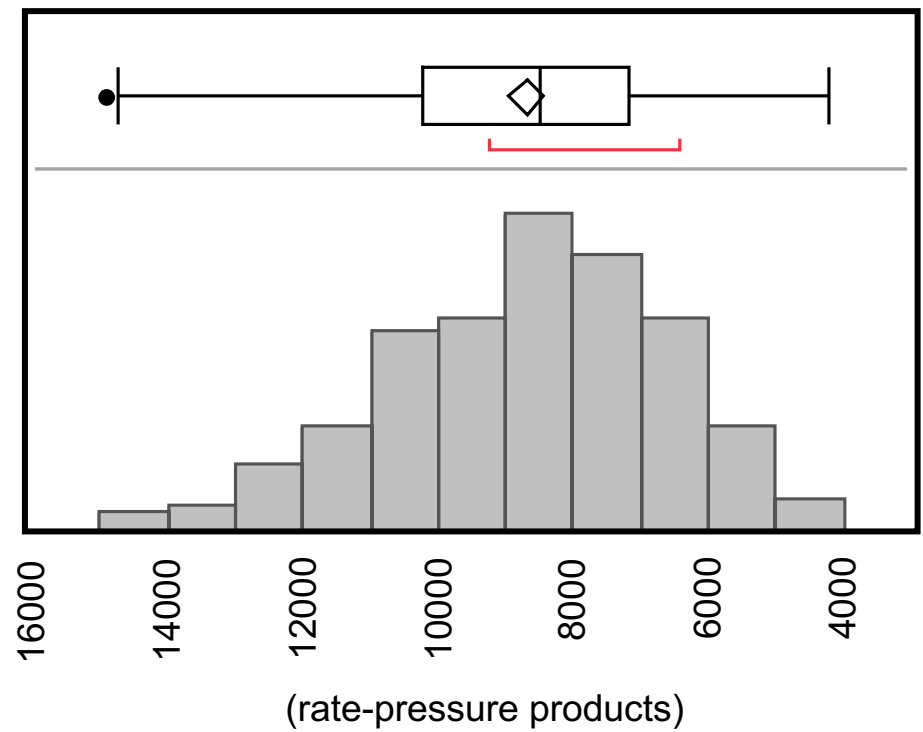

Supplement Figure 2

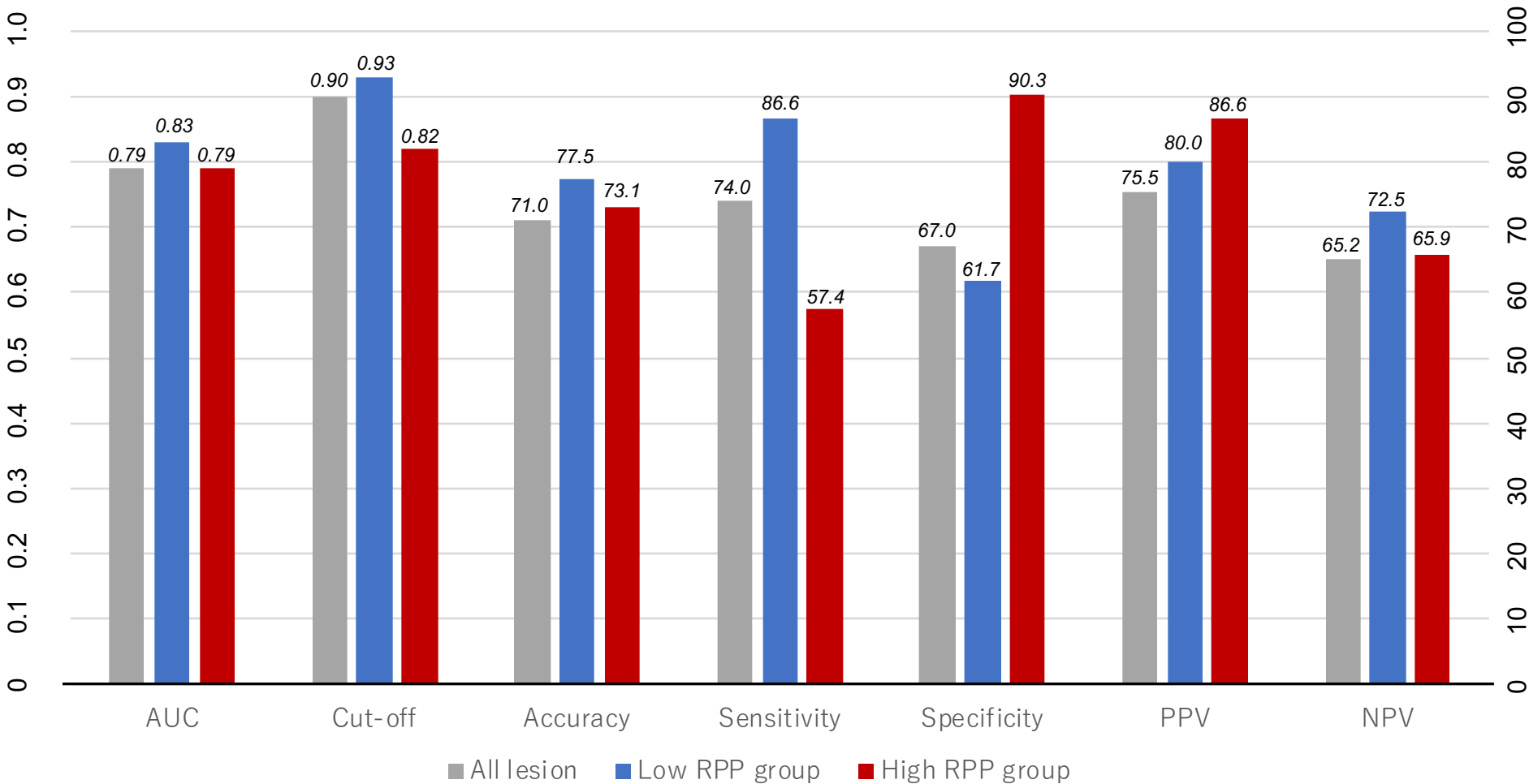

A) Low RPP group

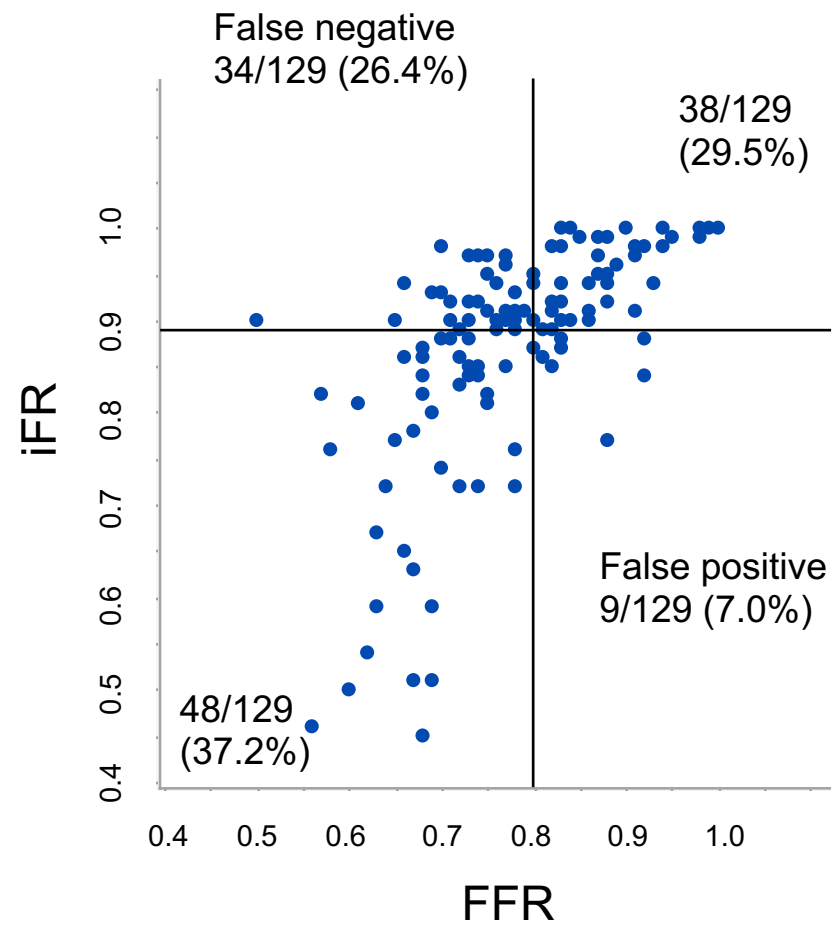

A) High RPP group

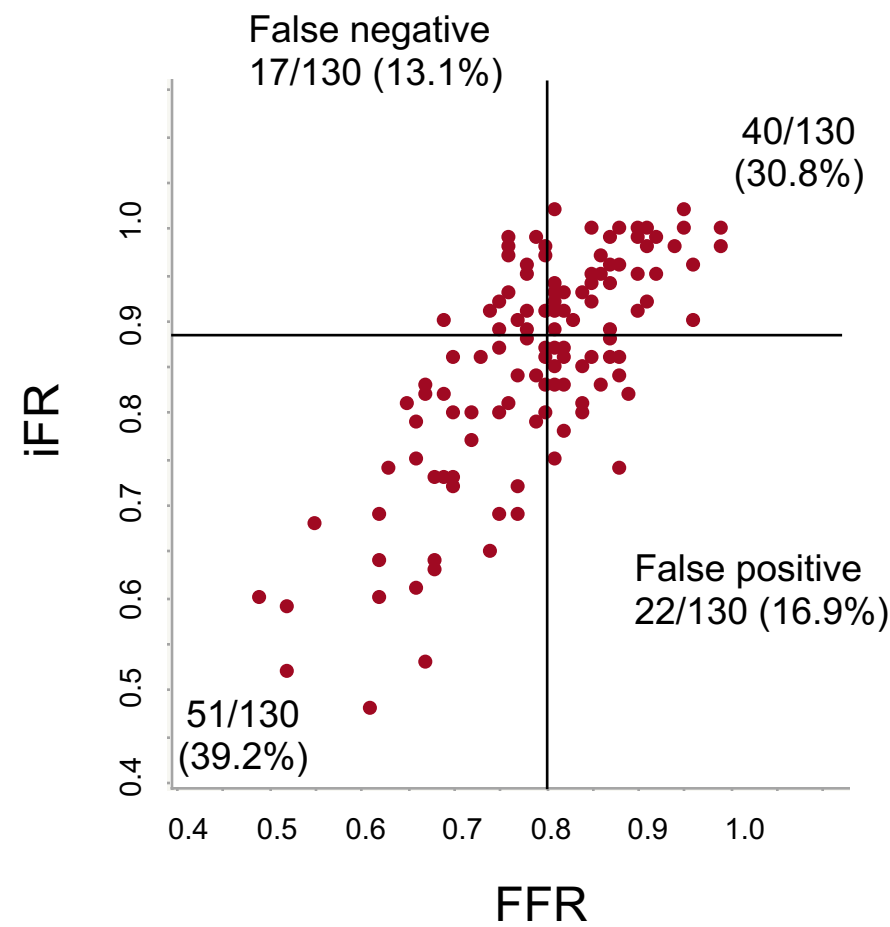

Supplement: Supplementary Materials — Supplemental Figure 1: distribution of the rate pressure products. Supplemental Figure 2: diagnostic ability of the iFR in the high-RPP and low-RPP groups with respect to the FFR. Supplemental Figure 3: correlation between the FFR and iFR in the high-RPP and low-RPP groups. [file 6230153.f1.pdf]
